# Definition of CD4 Immunosignatures Associated with MTB

**DOI:** 10.3389/fimmu.2014.00124

**Published:** 2014-03-24

**Authors:** Cecilia S. Lindestam Arlehamn, Alessandro Sette

**Affiliations:** ^1^Division of Vaccine Discovery, La Jolla Institute for Allergy and Immunology, La Jolla, CA, USA

**Keywords:** tuberculosis, T cells, epitope, HLA, genome-wide

## Abstract

We have recently described the first true genome-wide screen for CD4^+^ T-cell reactivity directed against *Mycobacterium tuberculosis* (MTB) in latent TB-infected individuals. The approach relied on predictions of HLA-binding capacity for a panel of DR, DP, and DQ alleles representative of those most commonly expressed in the general population, coupled with high throughput ELISPOT assays. The results identified hundreds of novel epitopes and antigens, and documented the novel observation that T cells in latent MTB infection are confined to the CXCR3^+^CCR6^+^ phenotype and largely directed against three antigenic “islands” within the MTB genome. In parallel, we have made generally available to the scientific community the technical approaches and reagents developed in the process, such as motifs, algorithms, and binding assays for several common HLA class II alleles, and a panel of single allele HLA class II transfected cell lines representative of the most frequent specificities in the general population. Recent efforts have been focused on characterization of epitopes and antigens recognized by patients with active TB and individuals vaccinated with BCG, with the aim of providing the first systematic evaluation of the overlap between latent, active, and BCG cohorts. The definition of a broad range of epitopes restricted by common HLA molecules, will facilitate development of diagnostic reagents, allow a rigorous evaluation of T-cell responses associated with TB infection in humans, and enable the evaluation of the immunogenicity of different vaccine candidates. Furthermore, it might suggest new candidates for vaccine and diagnostic development.

## TB as a Worldwide Medical Problem

Tuberculosis is the second leading cause of death from infectious diseases worldwide (1). The World Health Organization (WHO) estimates that approximately one-third of the world’s population (two billion total) is infected with *Mycobacterium tuberculosis* (MTB). MTB is responsible for 1.4 million deaths annually and 9 million new infections are reported each year. The majority of infected individuals control the pathogen by mounting a successful, long-lived, and protective immune response, leading to either resolution or a clinically latent infection. Approximately, 10% of latently infected individuals subsequently develop active TB (2, 3). The risk of developing active tuberculosis is higher in immunocompromised individuals (due to age, corticosteroids, malnutrition, HIV infection, etc.). Treatment is lengthy and expensive, requiring a combination of antibiotics. In many parts of the world, access to these drugs is limited and compliance with the drug regime is often poor, thus precipitating the development of drug-resistant strains. Worldwide, 3.7% of new cases and 20% of previously treated cases are infected with multidrug-resistant TB (MDR-TB), extensively drug-resistant TB (XDR-TB) and recently virtually untreatable totally drug-resistant (TDR) strains (1, 4). The prevalence of these drug-resistant cases, which complicates the schedule and increases cost of treatment, has heightened interest in the development of effective vaccines, and prompted inclusion of MTB in the list of A–C pathogens. The vaccination of children with *Mycobacterium bovis* BCG results in a 60–80% decrease in the incidence of active tuberculosis. However, in most developed countries BCG vaccination is not recommended due to the relatively low incidence of disease and variable effectiveness in preventing pulmonary TB in adults, a large fraction of active disease cases.

## CD4 T-Cell Responses in TB Infection

Due to the intracellular lifestyle of MTB, immunity relies on a successful T-cell response against a repertoire of antigenic targets. Defining this is central to understanding the immune response against TB and it has been vigorously pursued. Human T-cell responses to MTB involve CD4, CD8, CD1, and γδ T cells, though protective immunity to MTB is commonly ascribed to a Th1 profile (3, 5–8). CD4^+^ T cells are central to the defense against MTB, as exemplified by the fact that HIV-infected patients are more susceptible to primary TB infection, reinfection, and reactivation (9–11). Seminal studies in human T-cell responses to MTB showed that memory Th1 cells secreted IFNγ (12). It was further shown that IFNγ has an essential role in the protective immunity to mycobacteria, as individuals with genetic defects in the IFNγ receptor have an increased susceptibility to infection with mycobacteria (13). Furthermore, TNFα is important in host resistance to TB, as evidenced by studies following anti-TNFα therapy for autoimmune disease, where patients with LTBI have been observed to develop active TB (14, 15). Other Th subsets and cytokines have been shown to be involved in the T-cell response to MTB. Several studies indicate that lower IFNγ/IL-4 or IFNγ/IL-5 ratios are found in active TB patients compared to healthy TB controls (16, 17). Furthermore, healthcare workers that have worked in close proximity to TB patients, and subsequently developed TB, showed increased IL-5 levels compared to healthcare workers that did not develop TB (18). Definition of the exact role of Th2 responses still requires more investigation. Furthermore, several studies suggest that the capacity to secrete multiple cytokines can determine pathogen clearance versus persistence (19). Indeed, several studies in TB have suggested that multifunctional T cells are not only a potential correlate of protection, but have also been implicated in pathology (20–23). Recently, MTB-specific T-cell expression of IL-17 has been described (24–27). It has been demonstrated that the BCG vaccine and purified protein derivative (PPD) are able to expand memory CD4^+^IL-17^+^ cells (24, 26). Furthermore, IL-17^+^ T cells have been described in active TB patients, particularly in those infected by MDR MTB strains (27, 28), suggesting a pathogenic role for this cytokine. There is also evidence for IL-10 as a factor in humans with active TB, where IL-10 mediates inhibition of antigen presentation to T cells, and therefore mediates a decreased ability to clear infection contributing to TB pathogenesis (29). IL-10 has been shown to be elevated in serum from active pulmonary TB patients (30). Therefore, the involvement of different Th subsets in TB infection remains to be clarified and definition of human CD4^+^ T-cell epitopes to specifically track pathogen-specific T-cell subsets remains a high priority.

## The Concept of HLA Promiscuity

T cells recognize specific complexes formed between MHC molecules and particular peptide epitopes. Accordingly, a given epitope will elicit a T-cell response only in individuals expressing MHC molecules having the capacity to bind it with sufficiently high affinity. Both class I and class II MHC molecules are extremely polymorphic, and thousands of different variants are known in humans (31, 32). The most frequent HLA class II alleles with population coverage of almost 90% at each locus are shown in Table 1. Much of the polymorphism is concentrated on residues located in the peptide-binding groove, giving each allelic variant a distinct binding specificity. As a result, the prediction, identification, and validation of epitopes restricted by each HLA type represent tasks of such complexity as to be practically unfeasible. Further, different MHC types are expressed at dramatically different frequencies in and across different ethnicities. Thus, without careful consideration, ethnically unbiased population coverage is difficult to obtain.

**Table 1:** **Genotype and phenotype frequencies of HLA class II alleles**.

Locus	Allele	Genotype frequency	Phenotype frequency
DRB1	DRB1*01:01	2.8	5.4
	DRB1*03:01	7.1	13.7
	DRB1*03:02	1.1	2.1
	DRB1*04:01	2.3	4.6
	DRB1*04:02	1.1	2.2
	DRB1*04:03	2.3	4.5
	DRB1*04:04	1.9	3.8
	DRB1*04:05	3.1	6.2
	DRB1*04:07	2.4	4.8
	DRB1*04:11	1.6	3.3
	DRB1*07:01	7	13.5
	DRB1*08:02	2.5	4.9
	DRB1*09:01	3.1	6.2
	DRB1*11:01	6.1	11.8
	DRB1*11:02	1.1	2.2
	DRB1*11:03	0.3	0.5
	DRB1*11:04	1.4	2.8
	DRB1*12:01	2	3.9
	DRB1*13:01	3.2	6.3
	DRB1*13:02	3.9	7.7
	DRB1*13:03	1.2	2.4
	DRB1*13:04	0.1	0.2
	DRB1*14:01	3.4	6.7
	DRB1*14:02	2.8	5.6
	DRB1*15:01	6.3	12.2
	DRB1*16:01	1	1.9
DRB1	Total	71.1	91.7
DRB3/4/5	DRB3*01:01	14	26.1
	DRB3*02:02	18.9	34.3
	DRB3*03:01	6.7	13
	DRB4*01:01	23.7	41.8
	DRB5*01:01	8.3	16
	DRB5*01:02	5.1	9.8
DRB3/4/5	Total	76.7	94.6
DQA1/DQB1	DQA1*05:01/DQB1*02:01	5.8	11.3
	DQA1*02:01/DQB1*02:01	5.7	11.1
	DQA1*05:01/DQB1*03:01	19.5	35.1
	DQA1*03:01/DQB1*03:02	10	19
	DQA1*04:01/DQB1*04:02	6.6	12.8
	DQA1*01:01/DQB1*05:01	7.6	14.6
	DQA1*01:02/DQB1*05:02	3.5	6.9
	DQA1*01:02/DQB1*06:02	7.6	14.6
DQA1/DQB1	Total	66.3	88.7
DPA1/DPB1	DPA1*02:01/DPB1*01:01	8.4	16
	DPA1*01:03/DPB1*02:01	9.2	17.5
	DPA1*01:03/DPB1*04:01	20.1	36.2
	DPA1*01:03/DPB1*04:02	23.6	41.6
	DPA1*02:02/DPB1*05:01	11.5	21.7
	DPA1*02:01/DPB1*14:01	3.8	7.4
DPA1/DPB1	Total	76.5	94.5

*Average genotype and phenotype frequencies for individual alleles are based on data available at dbMHC, as previously described by McKinney et al. (33). Alleles previously characterized in detail for binding specificity are highlighted in bold (32)*.

One mean of circumventing the problem is to focus on the HLA types that are most widely represented in different ethnicities worldwide (Table 1), while at the same time selecting epitopes that are capable of binding multiple common HLA types (promiscuous epitopes). In this respect, it has been found that both class I and II HLA molecules can be classified into groups, denominated as supertypes that reflect shared or largely overlapping peptide-binding repertoires and specificities. Indeed, a large body of evidence demonstrates that the repertoire of peptides bound by different HLA class II molecules significantly overlap (32), and peptides with promiscuous binding capacity are also quite common [see, e.g., (34, 35)]. Overlaps in the repertoires of DRB1 and DRB3/4/5 molecules, leading to the definition of a DR supertype, as well as the identity of well-characterized CD4 T-cell epitopes with promiscuous DR-binding capacity, have been known for over a decade (34–36). Other studies have similarly addressed repertoire overlaps and the existence of corresponding supertypes for DP (32, 37–39) and DQ (32, 40, 41).

Following upon earlier computational, structural, and functional approaches to define class II supertypes (42–45), we utilized a large library of HLA DR-, DQ-, and DP-binding data to define seven different class II supertypes (main DR, DR4, DRB3, main DQ, DQ7, main DP, and DP2) (32). The molecules associated with the respective supertypes fell largely along lines defined by MHC locus and reflect, in broad terms, commonalities in reported peptide-binding motifs. Repertoire overlaps between molecules within the same class II supertype were found to be similar in magnitude to what has been observed for HLA class I supertypes. Surprisingly, however, the degree to which repertoires between molecules in the different class II supertypes overlapped was found to be fivefold to tenfold higher than repertoire overlaps typically noted between molecules in different class I supertypes. These results highlight the existence of a high degree of repertoire overlap amongst all HLA class II molecules, regardless of supertype association. Further, in terms of implications for epitope identification studies, these data also validate the idea that broadly reactive HLA class II epitopes can be defined.

### HLA promiscuity implications for epitope identification

Peptides with highly promiscuous binding capacity are frequently recognized by immune individuals (34, 46–49) and epitope immunodominance is highly influenced by promiscuous recognition in the context of multiple HLA class II molecules (50). Furthermore, a dominant fraction of the pathogen or allergen-specific response can be identified by selection of the most promiscuous binding peptides using bioinformatic predictions (51–54). The advantage of this approach is that it would identify the optimal set of peptide candidates for immunogenicity testing, eliminating the necessity of synthesizing a large number of overlapping peptides and, more importantly, circumvent the need to test each one of them for binding to numerous HLA class II molecules *in vitro*.

We have recently described the selection of a panel of HLA DR, DQ, and DP specificities that provide worldwide population (phenotypic) coverage of almost 90% at each locus, and accounts for over 66% of all genes at each locus (32) (Table 1). Considering up to eight different class II alleles expressed per individual (i.e., up to two at each of the four class II loci – DRB1, DRB3/4/5, DQ, and DP), this panel afforded coverage of at least four alleles in over 95% of the individuals in four different study populations of diverse ethnicity from the USA and South Africa (33). For each of these allelic variants, single HLA class II allele-transfected cell lines have been generated (33). These transfected cell lines can be used for high throughput determination of HLA restriction, enabling better characterization of T-cell responses, and facilitating the development of tetrameric staining reagents. Also, for the vast majority of these alleles high throughput binding assays have been established, peptide-binding motifs defined, and predictive algorithms developed and made publically available (32, 55), enabling efficient and thorough identification of candidate epitopes, as well as characterization of their HLA-binding capacity and potential population coverage.

Taken together, these data highlight that broadly reactive HLA class II epitopes can be identified, and that these promiscuous epitopes can account for a large fraction of the specific immune response. Further, the bioinformatic tools necessary to identify candidate epitopes, as well as specific cellular and immunochemical reagents to allow detailed characterization of epitope-specific responses are available and have been well-validated in several studies.

## Screen of a Genome-Wide Library of MTB-Derived Predicted HLA Class II Epitopes in LTBI Donors

The MTB genome encodes more than 4,000 different open reading frames (ORFs) (56), generally highly conserved amongst different strains, including drug-resistant ones. Identification of T-cell epitopes from such a large and complex target is a complex task, yet necessary for disease monitoring, vaccine evaluations, and development. A comprehensive genome-wide screen for HLA class II epitopes was recently performed (57). This genome-wide screen analyzed the reactivity of latent TB-infected (LTBI) individuals from the San Diego area. LTBIs were initially chosen as representative of a patient population that is, at least in part, capable of containing TB infection. Several 100 novel CD4-restricted epitopes and many antigens were identified (57). Furthermore, this study documented the novel observation that T cells in latent MTB infection are confined to the recently described CXCR3^+^CCR6^+^ phenotype (24, 58) and largely directed against three antigenic “islands” within the MTB genome. Still, important gaps in epitope knowledge remain, as also highlighted in the TB research community, in particular after the disappointing results of the MVA85A BCG boost human vaccine trial (59).

To enable a genome-wide screen for epitopes recognized by LTBIs (57), protein sequences from five complete (CDC1551, F11, H37Ra, H37Rv, and KZN 1435) MTB genomes and 16 draft assemblies available in the NCBI Protein database were aligned. To select candidate promiscuous epitopes, the binding capacity of all possible 15-mer peptides was predicted for 22 HLA DR, DP, and DQ class II alleles commonly expressed in the general population and for which validated algorithms were available (32, 52). This approach eliminates the need to test each peptide *in vitro* for HLA class II binding, as well as the necessity of synthesizing overlapping peptides. The resulting synthetic peptide library of 20,610 peptides (2–10 per ORF, average 5), were tested in high throughput *ex vivo* IFNγ ELISPOT using circulating T cells from LTBI donors. Each individual donor tested recognized 24 epitopes on average, revealing striking heterogeneity of responses to MTB. The epitopes identified were ranked on the basis of magnitude of response to assess their relative dominance. Overall, the top 80 epitopes accounted for 75% of the total response and the top 175 epitopes accounted for 90% of the total response. The epitopes were mapped to individual MTB antigens using the H37Rv as a reference genome. A total of 82 antigens were recognized by more than 10% of LTBI donors, accounting for approximately 80% of the total response. Thus, natural immunity to MTB is multiantigenic. Taken together, these results demonstrate the feasibility, novelty, and success of the genome-wide screen for epitopes recognized by LTBIs.

### Characteristics of HLA class II restricted antigens identified by the genome-wide approach

The protein category and the genomic location of the identified antigens were determined using the TubercuList database (60). This revealed enrichment for responses against cell wall-associated and secreted proteins, however, strong immune responses were induced by both secreted and non-secreted proteins, consistent with earlier antigen discovery efforts (61–63). The localization of antigens recognized by the LTBI donors was visualized by plotting the recognition data on a linear map of the MTB genome. This revealed striking clusters of reactivity within certain regions of the genome. In particular, three significant antigenic islands, which encode 0.55% of the total ORFs, accounted for 42% of the total response. All three islands were shown to contain ESX protein pairs, such as the well-known Rv3875 (Early Secretory Target-6, ESAT-6) and Rv3874 (Culture Filtrate Protein 10, CFP10) (64), and two also contain Type VII secretion systems ESX-1 and ESX-3.

### Methods to characterize and validate identified epitopes

Characterization and tracking of pathogen-specific T cells can be achieved once specific T-cell epitopes have been defined. Here, we briefly review our published data relating to the characterization of T cells derived from LTBI donors (57). A variety of approaches were employed in parallel including multiparameter intracellular cytokine staining (ICS) assays, tetramer staining, and T-cell libraries (65). It was found that CD4^+^ T cells recognizing epitopes derived from different TB antigens were associated with similar multifunctional cytokine expression patterns. The most frequent CD4^+^ T cells were IFNγ^+^TNFα^+^IL-2^+^ or IFNγ^+^TNFα^+^, followed by TNFα^+^ single producing CD4^+^ T cells. To a lesser extent, TNFα^+^IL-2^+^, single IFNγ^+^, and single IL-2^+^ cells were also detected.

To characterize the responding T cells in depth, HLA-epitope tetramer reagents were prepared for representative epitopes for staining of CD4^+^ purified cells. To overcome low T-cell frequency, a magnetic bead enrichment technique was preformed (50, 66). This allowed phenotypic characterization of epitope-specific memory subsets (57) as well as Th subset characterization (manuscript in preparation).

An alternative and complementary approach to ICS and tetrameric staining reagents is the screening of T-cell libraries (65). This high throughput method allows determination of frequency and distribution of pathogen/antigen/epitope-specific T cells (67).

## The Repertoire of T-Cell Epitopes in the Different Clinical Manifestations of MTB is Not Fully Defined

The work described above developed reagents and approaches to broadly characterize human T-cell epitopes in the general human population (32, 33, 39, 41, 68), and characterized in detail the T-cell epitopes recognized in a panel of model TB antigens (50). Most importantly, using LTBI donor PBMCs, we performed the first truly genome-wide screen of *ex vivo* human CD4^+^ MTB T-cell reactivity. Since latently infected individuals are able to control infection, they provided a logical relevant “first step” population to study protective responses. Mapping T-cell responses from these individuals identified immunodominant CD4^+^ T-cell antigens associated with potentially protective responses, and thus relevant to vaccine design.

According to most classifications, three primary and different outcomes can follow MTB exposure. The first, active TB infection is usually associated with evidence of bacterial replication. The second, LTBI, is usually associated with no disease symptoms and an effective immune response. And thirdly, reactivation of tuberculosis is often triggered by immunosuppression (69). MTB is believed to express different proteins in different stages of infection that may give rise to stage-specific immune responses and recognition of different antigens. Evidence of infection and stage-specific antigens in humans has indeed been reported (70, 71). In granulomas, MTB is believed to be in a dormant state, triggered by a range of stress factors including hypoxia, low pH, NO, nutrient deprivation, and host immune pressure (72). Under these conditions, genes encoded by the DosR regulon are upregulated (73, 74) and several antigens encoded by this regulon have been described as preferentially recognized by individuals with LTBI (71, 75–77). In addition, some proteins have been described and referred to as “resuscitation antigens” (78, 79). These are small bacterial proteins that promote proliferation of dormant mycobacteria, and are therefore believed to be involved in the reactivation of MTB (80). However, these antigens have not been described as being preferentially associated with a certain stage of infection. The availability of prediction methods and high throughput assays makes it possible to investigate genome-wide disease stage-specific TB reactivity and they can be applied to other pathogen systems.

## Vaccination Against TB: Need for Reappraisal of BCG

One of the long-term strategies essential for control of the global TB epidemic is effective vaccination. The only available licensed TB vaccine to date, BCG, protects against disseminated tuberculosis in young children but offers very variable protection against pulmonary tuberculosis (the contagious transmittable form of the disease) in children and adults (81–83).

Several candidate TB vaccines are in clinical trial and many of them are designed to boost the BCG response. One candidate is MVA85A (modified Vaccinia Ankara virus expressing antigen 85A; Rv3804c), which was developed as a heterologous boost for BCG (84, 85). Rv3804c is highly conserved amongst mycobacterial species and it is present in all strains of BCG (86). MVA85A was shown to boost pre-existing antimycobacterial immune responses induced by either environmental mycobacteria or BCG vaccination (84). A recent phase 2b study demonstrated the feasibility of a large efficacy trial of a new TB vaccine in a high-burden setting (59). This landmark trial was the first infant efficacy trial in over 50 years and its protocols and design will provide a reference for future trials and vaccination strategies. Unfortunately, however, the trial failed to show any efficacy against MTB infection in infants. Various hypotheses have been proposed to explain the lack of efficacy. Amongst them is the hypothesis that boosting with an antigen conserved in all mycobacterial species does not provide any additional benefit over the immunity already induced by exposure to environmental mycobacteria or BCG.

BCG was developed 90 years ago, but there is still a fundamental need for more knowledge regarding the actual mechanism of BCG immunogenicity, immunodominance, and crossreactivity with TB. Several studies have investigated mycobacterial antigen-specific human T-cell responses primed by vaccination with BCG (62, 87, 88). However, a genome-wide screen for epitopes has not previously been performed and would provide important immunological answers. First, it is imperative to clearly define the TB antigens that are primed following BCG vaccination. Identification of the antigens that are dominantly recognized following BCG vaccination and also recognized in natural TB infection would be important information for the design of BCG prime-boosting vaccines. Conversely, identification of antigens that are primed by BCG but are either weakly or not recognized in natural TB infection could provide new vaccination strategies, as elimination of such antigens may improve BCG efficacy. Second, the characterization of the T-cell phenotypes associated with recognition of various antigens in the context of BCG vaccination and natural infection may provide clues to the type of immune response that might be most desirable, and additionally indicate how the vaccine response should be modulated (i.e., by use of specific adjuvants). Finally, the identification and characterization of epitopes and antigens recognized following BCG vaccination would provide important tools to monitor and evaluate different vaccine candidates, or different vaccination strategies examining dose, route, and the use of different adjuvants. In conclusion, as it is likely that a large proportion of future vaccine recipients will be BCG immunized at birth, and because several new vaccine candidates aim at either replacing or augmenting the efficacy of the BCG vaccine (89–91), a thorough understanding of the immune response following BCG vaccination and in different disease stages of TB infection will provide much needed information toward future TB vaccine design and more efficacious predictive diagnostic tests.

## Conflict of Interest Statement

The authors declare that the research was conducted in the absence of any commercial or financial relationships that could be construed as a potential conflict of interest.
